# The clinical case report: a review of its merits and limitations

**DOI:** 10.1186/1756-0500-7-264

**Published:** 2014-04-23

**Authors:** Trygve Nissen, Rolf Wynn

**Affiliations:** 1 Department of Clinical Medicine, University of Tromsø, N-9038 Tromsø, Norway; 2 Division of General Psychiatry, University Hospital of North Norway, N-9291 Tromsø, Norway; 3 Division of Addictions and Specialized Psychiatry, University Hospital of North Norway, N-9291 Tromsø, Norway

**Keywords:** Case reports, Case series, Merits, Limitations, Methodology

## Abstract

**Background:**

The clinical case report has a long-standing tradition in the medical literature. While its scientific significance has become smaller as more advanced research methods have gained ground, case reports are still presented in many medical journals. Some scholars point to its limited value for medical progress, while others assert that the genre is undervalued. We aimed to present the various points of view regarding the merits and limitations of the case report genre. We searched Google Scholar, PubMed and select textbooks on epidemiology and medical research for articles and book-chapters discussing the merits and limitations of clinical case reports and case series.

**Results:**

The major merits of case reporting were these: Detecting novelties, generating hypotheses, pharmacovigilance, high applicability when other research designs are not possible to carry out, allowing emphasis on the narrative aspect (in-depth understanding), and educational value. The major limitations were: Lack of ability to generalize, no possibility to establish cause-effect relationship, danger of over-interpretation, publication bias, retrospective design, and distraction of reader when focusing on the unusual.

**Conclusions:**

Despite having lost its central role in medical literature in the 20th century, the genre still appears popular. It is a valuable part of the various research methods, especially since it complements other approaches. Furthermore, it also contributes in areas of medicine that are not specifically research-related, e.g. as an educational tool. Revision of the case report genre has been attempted in order to integrate the biomedical model with the narrative approach, but without significant success. The future prospects of the case report could possibly be in new applications of the genre, i.e. exclusive case report databases available online, and open access for clinicians and researchers.

## Background

Throughout history the clinical case report and case report series have been integral components of medical literature [1]. The case report genre held a strong position until it was sidelined in the second half of the 20^th^ century [2,3]. New methodologies for research articles paved the way for evidence-based medicine. Editors had to make space for these research articles and at the same time signaled less enthusiasm for publishing case reports [4]. This spurred some heated debates in medical journals as readers were worried that the traditional case report was in jeopardy [5,6]. Those who welcomed the new trend with fewer case reports being published pointed mainly to their low quality and inclination to emphasize mere curiosa [7-9]. Some of the proponents of the genre claimed that the case report had been and still was indispensible for furthering medical knowledge and that it was unique in taking care of the detailed study of the individual patient as opposed to the new research methods with their “…nomothetic approach [taking] precedence…” [5]. Still, the case report got a low ranking on the evidence hierarchy. After a decline in popularity a new interest for the case report emerged, probably beginning in the late 1990s [2]. A peer-reviewed ‘Case reports’ section was introduced in the *Lancet* in 1995 [10]. In 2007, the first international, Pubmed-listed medical journal publishing only case reports was established [11,12]. In the following years, several similar journals, for the most part online and open-access, have been launched.

The present debate is not so much focused on whether case reporting is obsolete or not. Some of the discussions after the turn of the century have been about adapting the case report genre to new challenges. One example is the suggestion of incorporating the narrative, i.e. “… stressing the patient’s story”, in the case report [13]. The authors termed their initiative “The storied case report”. Their endeavor was not met with success. In analyzing the causes for this, they wondered if “… junior trainees find it too hard to determine what is relevant and senior trainees find it too hard to change their habits” [13]. A similar attempt was done when the editors of the *Journal of Medical Case Reports* in 2012 encouraged authors to include the patients’ perspectives by letting patients describe their own experiences [14].

Notwithstanding, we feel there is much to be gained from having an ongoing discussion highlighting the indications and contraindications for producing case reports. This can to some degree be facilitated by getting an understanding of the merits and limitations of the genre. The objective of this article is to present the merits and limitations of case reports and case series reports.

## Methods

We adopted *Taber’s Cyclopedic Medical Dictionary’s* definition of the *case report*: “A formal summary of a unique patient and his or her illness, including the presenting signs and symptoms, diagnostic studies, treatment course and outcome” [15]. A case report consists of one or two cases, most often only one. The *case series* or *case series report* usually consists of three to ten cases [16]. (In the following we use the term case report to denote both case reports and case series report). Case reports are most often naturalistic and descriptive. Sometimes, however, they can be prospective and experimental.

As literature specifically dealing with the case report genre seemed harder to elicit from the databases than the vast amount of particular case reports, we performed iterative searches. We searched Google Scholar and PubMed using the search terms ‘case report(s)’, ‘case series’, ‘case series report(s)’, ‘case reporting’ in various combinations with ‘clinical’, ‘medical’, ‘anecdotal’, ‘methodology’, ‘review’, ‘overview’, ‘strengths’, ‘weaknesses’, ‘merits’, and ‘limitations’. Further references were identified by examining the literature found in the electronic searches. We also consulted major textbooks on epidemiology [17,18], some scholars of medical genres [19,20] and a monograph on case reporting by the epidemiologist M. Jenicek [16]. We delimited our review to the retrospective, naturalistic, and descriptive case report, also labeled the “traditional” or “classic” case report, and case series including such reports. Thus we excluded other types, such as the planned, qualitative case study approach [21] and simulated cases [22-24]. Finally, we extracted the relevant data and grouped the merits and limitations items in rank order with the items we judged to be the most important first.

## Results

### Merits

#### New observations

The major advantage of case reporting is probably its ability to detect novelties [16]. It is the only way to present unusual, uncontrolled observations regarding symptoms, clinical findings, course of illness, complications of interventions, associations of diseases, side effects of drugs, etc. In short, anything that is rare or has never been observed previously might be important for the medical community and ought to be published. A case report might sensitize readers and thus facilitate detection of similar or identical cases.

#### Generating hypotheses

From a single, or preferably several single case reports or a case series, new hypotheses could be formulated. These could then be tested with formal research methods that are designed to refute or confirm the hypotheses, i.e. comparative (observational and experimental) studies.

There are numerous examples of new discoveries or major advancements in medicine that started with a case report or, in some cases, as humbly as a letter to the editor. The first concern from the medical community about the devastating side effect of thalidomide, i.e. the congenital abnormalities, appeared as a letter to the editor in the *Lancet* in 1961 [25]. Soon thereafter, several case reports and case series reports were published in various journals. Case reporting is thus indispensable in drug safety surveillance (pharmacovigilance) [26].

Sometimes significant advancements in knowledge have come not from what researchers were pursuing, but from “accidental discoveries”, i.e. by serendipity. The story of Alexander Fleming’s discovery of penicillin in 1928 is well known in the medical field [27]. Psychiatry has profited to a large degree from this mode of advancing medical science as many of the drugs used for mental disorders have been discovered serendipitously [27]. One notable example is the discovery of the effect of lithium on manic episodes in patients with manic-depressive disorder [28]. A more recent discovery is the successful treatment of infantile hemangiomas with systemic propranolol. This discovery was published, as a case series report, in the correspondence section in *New England Journal of Medicine*[29]. However, the evidence for the effect of this treatment is still preliminary, and several randomized trials are under way [30,31].

Clear and operational entities are prerequisites for doing medical research. Descriptions must come before understanding. Clinical observations that lead to new disorders being described are well suited for case reporting. The medical literature is replete with case-based articles describing new diseases and syndromes. One notable example is the first description of neurasthenia by G. Beard in *Boston Medical and Surgical Journal* in 1869 [32].

#### Researching rare disorders

For rare disorders randomized controlled trials (RCTs) can be impossible to run due to lack of patients to be enrolled. Research on drug treatment and other kinds of interventions must therefore be based on less rigorous methodologies, among them case series and case reports. This would be in accordance with the European Commission’s recommendation to its members to improve health care for those with rare disorders [33].

#### Solving ethical constraints

Case reporting can be valuable when ethical constraints prohibit experimental research. Take as an example the challenge of how to manage the side effects of accidental extravasation of cytotoxic drugs. As RCTs on humans seem unethical in this clinical situation the current guidelines rest on small observational studies, case reports and animal studies [34]. Or another example: Physical restraint is sometimes associated with sudden, unexpected death. The cause or causes for this are to some degree enigmatic, and it is hard to conceive of a controlled study that could be ethical [35,36]. Case reports and case series being “natural experiments” might be the only evidence available for guiding clinical practice.

#### In-depth narrative case studies

Case reporting can be a way of presenting research with an idiographic emphasis. As contrasted to nomothetic research, an idiographic approach aims at in-depth understanding of human phenomena, especially in the field of psychology and psychiatry. The objective is not generalizable knowledge, but an understanding of meaning and intentionality for an individual or individuals. Sigmund Freud’s case studies are relevant examples. This usage of case reports borders on qualitative research. Qualitative studies, although developed in the social sciences, have become a welcome contribution within health sciences in the last two decades.

#### Educational value

Clinical medical learning is to a large degree case-based. Typical case histories and vignettes are often presented in textbooks, in lectures, etc. Unusual observations presented as published case reports are important as part of doctors’ continuing medical education, especially as they demonstrate the diversity of manifestations both within and between medical diseases and syndromes [37,38]. Among the various medical texts, the case report is the only one that presents day-to-day clinical practice, clinicians’ diagnostic reasoning, disease management, and follow-up. We believe that some case reports that are written with the aim of contributing to medical knowledge turn out to be of most value educationally because the phenomena have already been described elsewhere. Other case reports are clearly primarily written for educational value [37]. Some journals have regular sections dedicated to educational case reports, e.g. The Case Records of the Massachusetts General Hospital in the *New England Journal of Medicine* and the Clinical Case Conference found in the *American Journal of Psychiatry.*

#### Expenses

The cost of doing a case report is low compared to planned, formal studies. Most often the necessary work is probably done in the clinical setting without specific funding. Larger studies, for instance RCTs, will usually need an academic setting.

#### Fast publication

The time span from observation to publication can be much shorter than for other kinds of studies. This is obviously a great advantage as a case report can be an important alert to the medical community about a serious event. The unexpected side effects of the sedative-antinauseant thalidomide on newborn babies is a telling story. The drug had been prescribed during pregnancy to the babies’ mothers. After the first published observation of severe abnormalities in babies appeared as a letter to the editor of the *Lancet* in December 16^th^, 1961 [25], several case reports and series followed [39,40]. It should be mentioned though that the drug company had announced on December 2^nd^, 1961, i.e. two weeks before the letter from McBride [25], that it would withdraw the drug form the market immediately [41].

#### Flexible structure

Riaz Agha, editor of the *International Journal of Surgery Case Reports* suggests that the case report, with its less rigid structure is useful as it “… allows the surgeon(s) to discuss their diagnostic approach, the context, background, decision-making, reasoning and outcomes” [42]. Although the editor is commenting on the surgical case report, the argument can be applied for the whole field of clinical medicine. It should be mentioned though, that other commentators have argued for a more standardized, in effect more rigid, structure [43].

#### Clinical practice can be changed

Case reporting can lead to or contribute to a change in clinical practice. A drug might be withdrawn from the market. Or a relabeling might change the attitude to and treatment of a condition. During Word War I the shell shock syndrome was labeled and described thoroughly in several articles in the *Lancet*, the first of them appearing in February 1915 [44]. The author was the British captain and military doctor Charles S. Myers. Before his efforts to bring good care and treatment to afflicted soldiers there had been a common misconception that many of these dysfunctional soldiers were malingerers or cowards.

#### Exercise for novice researchers

The case report format is well suited for young doctors not yet trained as researchers. It can be an opportunity for a first exercise in authoring an article and a preparation for a scientific career [37,45,46].

#### Communication between the clinical and academic fields

Articles authored by clinicians can promote communication between practicing clinicians and academic researchers. Observations published can generate ideas and be a trigger for further studies. For instance, a case series consisting of several similar cases in a short period can make up the case-group for a case–control study [47]. Clinicians could do the observation and publish the case series while the case–control study could be left to the academics.

#### Entertainment

Some commentators find reading case reports fun. Although a rather weak argument in favor of case reporting, the value of being entertained should not be dismissed altogether. It might inspire physicians to spend more time browsing and reading scientific literature [48].

#### Studying the history of medicine

Finally, we present a note on a different and unintended aspect of the genre. The accumulated case reports from past eras are a rich resource for researching and understanding medical history [49,50]. A close study of old case reports can provide valuable information about how medicine has been practiced through the centuries [50,51].

### Limitations

#### No epidemiological quantities

As case reports are not chosen from representative population samples they cannot generate information on rates, ratios, incidences or prevalences. The case or cases being the numerator in the equation, has no denominator. However, if a case series report consists of a cluster of cases, it can signal an important and possibly causal association, e.g. an epidemic or a side effect of a newly marketed drug.

#### Causal inference not possible

Causality cannot be inferred from an uncontrolled observation. An association does not imply a cause-effect relationship. The observation or event in question could be a mere coincidence. This is a limitation shared by all the descriptive studies [47]. Take the thalidomide tragedy already mentioned as an example; Unusual events such as congenital malformations in some of the children born to mothers having taken a specific drug during pregnancy does not prove that the drug is the culprit. It is a mere hypothesis until further studies have either rejected or confirmed it. Cause-effect relationships require planned studies including control groups that to the extent possible control for chance, bias and confounders [52].

#### Generalization not possible

From the argument above, it follows that findings from case reports cannot be generalized. In order to generalize we need both a cause-effect relationship and a representative population for which the findings are valid. A single case report has neither. A case series, on the other hand, e.g. many “thalidomide babies” in a short time period, could strengthen the suspicion of a causal relationship, demanding further surveillance and research.

#### Bias

Publication bias could be a limiting factor. Journals in general favor positive-outcome findings [53]. One group of investigators studying case reports published in the *Lancet* found that only 5% of case reports and 10% of case series reported treatment failures [54]. A study of 435 case reports from the field of dentistry found that in 99.1%, the reports “…clearly [had] a positive outcome and the intervention was considered and described as successful by the authors” [55].

#### Overinterpretation

Overinterpretation or misinterpretation is the tendency or temptation to generalize when there is no justification for it. It has also been labeled “the anecdotal fallacy” [56]. This is not a shortcoming intrinsic to the method itself. Overinterpretation may be due to the phenomenon of case reports often having an emotional appeal on readers. The story implicitly makes a claim to truth. The reader might conclude prematurely that there is a causal connection. The phenomenon might be more clearly illustrated by the impact of the clinician’s load of personal cases on his or her practice. Here exemplified by a young doctor’s confession: “I often tell residents and medical students, ‘The only thing that actually changes practice is adverse anecdote.’” [57].

#### Emphasis on the rare

As case reporting often deals with the rare and atypical, it might divert the readers’ attention from common diseases and problems [58].

#### Confidentiality

Journals today require written informed consent from patients before publishing case reports. Both authors and publishers are responsible for securing confidentiality. A guarantee for full confidentiality is not always possible. Despite all possible measures taken to preserve confidentiality, sometimes the patient will be recognized by someone. This information should be given to the patient. An adequately informed patient might not consent to publication. In 1995 in an Editorial in the *British Journal of Psychiatry* one commentator, Isaac Marks, feared that written consent would discourage case reports being written [59]. Fortunately, judged form the large number of reports being published today, it seems unlikely that the demand for consent has impeded their publication.

#### Other methodological limitations

Case reports and series are written after the relevant event, i.e. the observation. Thus, the reports are produced retrospectively. The medical record might not contain all relevant data. Recall bias might prevent us from getting the necessary information from the patient or other informants such as family members and health professionals.

It has also been held against case reporting that it is subjective. The observer’s subjectivity might bias the quality and interpretation of the observation (i.e. information bias).

Finally, the falsification criterion within science, which is tested by repeating an experiment, cannot be applied for case reports. We cannot design another identical and uncontrolled observation. However, unplanned similar “experiments” of nature can be repeated. Several such observations can constitute a case series that represents stronger indicative evidence than the single case report.

## Discussion

The major advantages of case reporting are the ability to make new observations, generate hypotheses, accumulate scientific data about rare disorders, do in-depth narrative studies, and serve as a major educational tool. The method is deficient mainly in being unable to deliver quantitative data. Nor can it prove cause-effect relationship or allow generalizations. Furthermore, there is a risk of overinterpretation and publication bias.

The traditional case report does not fit easily into the qualitative-quantitative dichotomy of research methods. It certainly shares some characteristics with qualitative research [16], especially with regard to the idiographic, narrative perspective – the patient’s “interior world” [60] – that sometimes is attended to. Apart from “The storied case report” mentioned in the Background-section, other innovative modifications of the traditional case report have been tried: the “evidence-based case report” [61], the “interactive case report” [62] and the “integrated narrative and evidence based case report” [63]. These modifications of the format have not made a lasting impact on the way case reports in general are written today.

The method of case reporting is briefly dealt with in some textbooks on epidemiology [17,18]. Journals that welcome case reports often put more emphasis on style and design than on content in their ‘instruction to authors’ section [64]. As a consequence, Sorinola and coworkers argue for more consensus and more consistent guidance on writing case reports [64]. We feel that a satisfactory amount of guidance concerning both style and content now exists [12,16,65,66]. The latest contribution, “The CARE guidelines”, is an ambitious endeavor to improve completeness and transparency of reports [66]. These guidelines have included the “Patient perspective” as an item, apparently a bit half-heartedly as this item is placed after the Discussion section, thus not allowing this perspective to influence the Discussion and/or Conclusion section. We assume this is symptomatic of medicine’s problem with integrating the biomedical model with “narrative-based medicine”.

In recent years the medical community has taken an increased interest in case reports [2], especially after the surge of online, exclusive case report journals started in 2007 with the *Journal of Medical Case Reports* (which was the first international, Pubmed-listed medical journal publishing only case reports) as the first of this new brand. The climate of skepticism has been replaced by enthusiasm and demand for more case reports. A registry for case reports, Cases Database, was founded in 2012 [67]. On the condition that it succeeds in becoming a large, international database it could serve as a register being useful for clinicians at work as well as for medical research on various clinical issues. Assuming Pamela P. Powell’s assertion that “[a]lmost all practicing physicians eventually will encounter a case worthy of being reported” [60] is valid, there should be no shortage of potential cases waiting to be reported and filed in various databases, preferably online and open access.

### Limitations of this review

There are several limitations to this study. It is a weakness that we have not been able to review all the relevant literature. The number of publications in some way related to case reports and case report series is enormous, and although we have attempted to identify those publications relevant for our purpose (i.e. those that describe the merits and limitations of the case report genre), we might have missed some. It was difficult to find good search terms for our objective. Still, after repeated electronic searches supplemented with manual searches in reference lists, we had a corpus of literature where essentially no new merits or limitations emerged.

As we point out above, the ranking of merits and limitations represents our subjective opinion and we acknowledge that others might rank the importance of the items differently.

The perspective on merits and limitations of case reporting has been strictly medical. As a consequence we have not analyzed or discussed the various non-medical factors affecting the publication of case reports in different medical journals [2]. For instance, case reports are cited less often than other kinds of medical research articles [68]. Thus they can lower a journal’s impact factor, potentially making the journal less attractive. This might lead some high-impact journals to publish few or no case reports, while other journals have chosen to specialize in this genre.

## Conclusions

Before deciding on producing a case report or case series based on a particular patient or patients at hand, the observant clinician has to determine if the case report method is the appropriate article type. This review could hopefully assist in that judgment and perhaps be a stimulus to the continuing debate in the medical community on the value of case reporting.

## Competing interests

The authors declare that there are no competing interests.

## Authors’ contributions

TN contributed to the conception, drafting, and revision of the article. RW contributed to the conception, drafting, and revision of the article. Both authors approved the final manuscript.
